# Editorial: Education in dermatology

**DOI:** 10.3389/fmed.2024.1391839

**Published:** 2024-04-03

**Authors:** Alejandro Molina-Leyva, Salvador Arias-Santiago

**Affiliations:** ^1^Junta de Andalucía, Seville, Spain; ^2^Virgen de las Nieves University Hospital, Granada, Spain

**Keywords:** education, teaching, dermatology, teledermatology, technology

Dermatology, the study of skin, hair, and nails, encompasses a vast and intricate web of diagnoses and treatments. As technology evolves and healthcare landscapes shift, so too does the need for dynamic and effective education in this specialized field. This editorial serves as an invitation to delve into the ever-evolving tapestry of dermatology education, ignited by the diverse research articles presented.

Our journey begins with understanding the perceptions of medical students toward teledermatology as an educational tool. This study unveils valuable insights into how this technology, often lauded for its efficiency and accessibility, is perceived by future dermatologists. The exploration continues with a closer look at the challenges and opportunities presented by fully online learning in dermatology education. This retrospective analysis sheds light on the potential hurdles and successes associated with embracing virtual classrooms in this hands-on field (Meng et al.; Ureña-Paniego et al.).

Moving beyond traditional learning methods, we then navigate the realm of medical students' evolving interest in research. This investigation delves into the changing trends and motivations driving students toward research within the field of dermatology, offering valuable insights into the future of the specialty (Sanabria-de la Torre et al.).

But the exploration doesn't stop there. The editorial introduces an intriguing analysis of ChatGPT's potential and limitations in dermatology. This study prompts us to consider the role of emerging technologies like AI in dermatological education, urging a critical evaluation of their true utility amidst the hype (Zhang et al.).

Finally, we delve into the crucial topic of resident training, examining the perspectives of dermatologists and residents themselves. This research sheds light on the experiences, challenges, and potential improvements within residency programs, ensuring future dermatologists are equipped with the necessary skills and knowledge (Li et al.; Porriño-Bustamante et al.).

These diverse research articles collectively paint a multifaceted picture of the current landscape of dermatology education. They ignite conversations about the integration of technology, the evolving needs of learners, and the constant pursuit of improvement within residency programs. As we move forward, it is crucial to continue exploring, innovating, and collaborating to ensure dermatology education remains dynamic, effective, and adaptable to the ever-changing healthcare landscape.

Dermatology education has important future challenges. Maintain attractive face-to-face teaching, incorporate teaching innovation strategies that contemplate artificial intelligence, teledermatology, new diagnostic support tools and research training for students.

Teaching based on the acquisition of skills and not the passive transmission of knowledge and the training of teachers and providing an adequate environment for the teaching-learning process to be carried out successfully is another challenge.

This editorial serves as an invitation to join the discourse, delve deeper into the presented research, and actively contribute to shaping the future of dermatology education. Let us collectively ensure that future generations of dermatologists are equipped with the knowledge, skills, and passion to excel in this ever-evolving field.

## Author contributions

AM-L: Conceptualization, Data curation, Formal analysis, Funding acquisition, Investigation, Methodology, Project administration, Resources, Software, Supervision, Validation, Visualization, Writing – original draft, Writing – review & editing. SA-S: Conceptualization, Data curation, Formal analysis, Funding acquisition, Investigation, Methodology, Project administration, Resources, Software, Supervision, Validation, Visualization, Writing – original draft, Writing – review & editing.

